# The Role of PSMA PET Imaging in Prostate Cancer: Current Applications and Future Directions

**DOI:** 10.1007/s11934-025-01268-2

**Published:** 2025-05-31

**Authors:** Raeesa Islam, Shrijal Desai, Melissa Moran, David M. Golombos

**Affiliations:** 1 Section of Urologic Oncology, Rutgers Cancer Institute of New Jersey and Rutgers Robert Wood Johnson Medical School, 285 George St. Apt 906, New Brunswick, NJ USA; 2 Philadelphia College of Osteopathic Medicine, Philadelphia, PA USA

**Keywords:** PSMA, Prostate cancer, PET/CT, Staging, Biochemical recurrence, Theranostics

## Abstract

**Purpose of Review:**

Prostate-specific membrane antigen (PSMA)-targeted positron emission tomography (PET) imaging has revolutionized prostate cancer detection and management. This review aims to evaluate the latest advancements in PSMA PET imaging, assess its clinical applications in various disease stages, and compare it to conventional imaging techniques. We sought to determine how PSMA PET impacts clinical decision-making, treatment strategies, and patient outcomes, with a focus on its expanding role in precision oncology.

**Recent Findings:**

Recent studies have demonstrated that PSMA PET exhibits superior sensitivity and specificity in detecting prostate cancer metastases, particularly in cases of biochemical recurrence and early-stage disease. The advent of radiolabeled PSMA ligands, such as 68Ga-PSMA-11 and 18 F-DCFPyL, has led to improved diagnostic accuracy. Furthermore, PSMA-targeted radioligand therapies (e.g., 177Lu-PSMA-617) show promising results in metastatic castration-resistant prostate cancer (mCRPC), offering a novel therapeutic avenue. Studies have also highlighted the role of PSMA PET in refining treatment plans, including guiding surgery and radiotherapy.

**Summary:**

PSMA PET imaging represents a paradigm shift in prostate cancer diagnostics, staging, and treatment monitoring. It has led to earlier and more accurate detection of metastases, significantly altering management strategies. The growing body of evidence supports its integration into standard-of-care protocols, with ongoing research focusing on optimizing its therapeutic applications. Future studies should explore cost-effectiveness, accessibility, and potential synergies with emerging systemic therapies to further enhance patient outcomes.

## Introduction

Prostate cancer is a major public health concern, ranking as the second leading cause of cancer-related deaths in men, with an estimated 288,300 new cases and 34,700 deaths in the U.S. in 2023 [1]. Its disease course varies widely, highlighting the need for accurate and timely diagnosis. Conventional imaging, including multiparametric MRI (mpMRI), computed tomography (CT), and bone scintigraphy, has long been used for diagnosis and staging. mpMRI is particularly valuable for assessing tumor characteristics such as extracapsular extension and seminal vesicle invasion [2]. However, these conventional methods often fail to detect small metastases or local recurrences, especially in cases of biochemical recurrence (BCR), where PSA levels rise without clear imaging findings [3]. This limitation has driven interest in molecular imaging techniques offering greater sensitivity and specificity.

Prostate-specific membrane antigen (PSMA) has emerged as a critical biomarker in prostate cancer detection and management. A transmembrane protein that is overexpressed in prostate cancer cells, PSMA serves as an ideal target for imaging and therapy [4]. The advent of PSMA-targeted imaging agents, particularly Gallium-68-labeled tracers (68Ga-PSMA), has revolutionized prostate cancer diagnostics, first gaining traction in Europe about a decade ago [5]. 68Ga-PSMA positron-emission tomography (PET)/CT has shown superior sensitivity and specificity, particularly in detecting BCR and high-risk disease [6]. Among tracers, 68Ga-PSMA-11 is now a standard for PET imaging in prostate cancer, while fluorine-18-based tracers like 18 F-DCFPyL have demonstrated comparable efficacy [7].

Both 68Ga-PSMA and 18 F-DCFPyL have received FDA approval, marking a significant advancement in prostate cancer diagnostics. These agents enhance diagnostic accuracy and improve treatment planning [8]. The integration of PSMA PET imaging into clinical practice has been transformative, especially for biochemical recurrence cases where conventional imaging is often inadequate [9]. A prospective study showed that PSMA PET imaging led to significant changes in patient management by identifying previously undetected metastases [10].

Beyond diagnosis and staging, PSMA PET imaging is valuable for monitoring treatment response and detecting recurrence, offering insights into tumor biology and therapeutic efficacy [11]. As prostate cancer management evolves, PSMA PET imaging is set to play an increasingly central role, providing clinicians with powerful tools to enhance patient outcomes. The approval and adoption of 68Ga-PSMA and 18 F-DCFPyL reflect a pivotal shift in imaging strategies, underscoring the need for continued research into optimizing these techniques for personalized cancer care.

## Current Clinical Applications

### Initial Staging and Risk Stratification

In patients with high-risk localized prostate cancer, accurate staging is critical for treatment planning. Conventional imaging has limited sensitivity in detecting nodal and distant metastases, with mpMRI sensitivity for pelvic lymph node (PLN) involvement ranging from 39 to 75% [3].

PSMA PET/CT has demonstrated superior accuracy in primary staging. A prospective study comparing 68Ga-PSMA-11 PET/CT and conventional imaging in high-risk prostate cancer found that PSMA PET/CT detected metastases in 27% of cases previously classified as localized, leading to treatment modifications in 28% of patients [12].

### Biochemical Recurrence

Biochemical recurrence (BCR), defined as a rising PSA after radical prostatectomy or radiotherapy, poses a significant clinical challenge. Traditional imaging often fails to localize recurrence at low PSA levels. PSMA PET/CT has significantly improved the detection of recurrent disease. A a study comparing the utilization of multiparametric MRI with 68Ga-PSMA PET/CT identified that multiparametric MRI missed the detection of lesions with a diameter smaller than 1 cm, even in the cases of high grade lesions [13]. However, 68Ga -PSMA PET imaging is reliable even when the Gleason score is 6 or less, and therefore may be more promising compared to conventional imaging [13].

Furthermore, PSMA PET/CT led to a change in management in 62% of patients with BCR, including modifications in salvage radiotherapy planning and systemic therapy decisions [14]. The higher sensitivity at low PSA levels enables earlier intervention and opening the door for potential metastasis directed threapy, potentially improving long-term outcomes by targeting early recurrence before further progression.

### Advanced/Castrate-Resistant Disease

In castration-resistant prostate cancer (CRPC), conventional imaging often underestimates disease burden, particularly in patients with micrometastatic or oligometastatic disease. PSMA PET/CT has emerged as an essential tool in CRPC management, demonstrating superior sensitivity in detecting small-volume metastases and monitoring treatment response.

The role of PSMA imaging in CRPC extends beyond diagnostic capabilities to therapeutic applications. New PSMA detected lesions often lead to changes in systemic therapy. In addition, PSMA-targeted radioligand therapy represents a significant advancement in treatment options for metastatic CRPC. The VISION trial demonstrated that Lutetium-177 (177Lu)-PSMA therapy, guided by PSMA PET imaging, led to a 38% reduction in the risk of death compared to standard of care [12]. This landmark study established PSMA theranostics as a viable treatment option for patients with limited therapeutic alternatives.

Furthermore, PSMA imaging helps identify patients most likely to benefit from radioligand therapy. Studies have shown that higher PSMA expression on PET imaging correlates with better treatment responses to 177Lu-PSMA therapy [15]. This allows for better patient selection and potentially improved outcomes through personalized treatment approaches.

## Role in Local Disease Assessment

### Comparison with mpMRI

MRI has long been the gold standard for localized prostate cancer assessment due to its superior soft tissue characterization. However, emerging evidence suggests that PSMA PET may offer distinct advantages in specific clinical scenarios [9]. A meta-analysis by de Rooji et al. found that MRI had an overall stage T3 detection sensitivity of only 0.61 (95% CI 0.54–0.67), which improved only slightly with T2-weighted imaging and higher field strengths.

The features that MRI assesses include obliteration of the rectoprostatic angle, neurovascular bundle asymmetry, and seminal vesical asymmetry, which are relatively late markers of EPE and may only be visible when the tumor is already advanced [16]. The low sensitivity of MRI for T3 disease is not likely to be due to a radiological learning curve [17]. MRI detection of EPE could potentially be improved if radiologists estimated the likelihood of EPE rather than the currently implemented binary system of reporting whether EPE is present or not [16].

PSMA PET/CT has demonstrated superior detection of locally advanced tumors compared to mpMRI. In a recent study, the sensitivity for extracapsular extension (ECE) was 90.2% with PSMA PET/CT versus 60.0% with mpMRI, though mpMRI maintained a slightly higher specificity of 83.1% compared to 76.2% [18]. Importantly, there were no differences between two PSMA-targeted PET radiopharmaceuticals, 68Ga-PSMA-11 and 68Ga-P16-093 [18].

### Detection of EPE and SVI (T3 disease)

The assessment of extracapsular extension (ECE) and seminal vesicle invasion (SVI) is crucial for determining appropriate therapeutic approaches. Studies have shown excellent detection rates with PSMA PET/CT, reporting sensitivity of 0.90, specificity of 0.90, positive predictive value of 0.90, and negative predictive value of 0.90 for EPE [19]. For SVI, the metrics were similarly impressive: sensitivity of 0.75, specificity of 1.00, PPV 1.00, and NPV 0.97.

Interestingly, Dinckal et al. found contrasting results, with mpMRI showing higher sensitivity when detecting EPE (0.83 versus 0.44 with PSMA PET/CT) and bladder neck invasion (BNI) (sensitivity 0.29 and specificity 0.99 with mpMRI; 0.17 and 0.88 with PSMA PET/CT) [20]. However, PSMA PET/CT was superior at detecting SVI (sensitivity 0.75; 0.55 with mpMRI) and lymph node metastases (sensitivity 0.50; 0.30 with mpMRI) [20].

### Impact on Surgical Planning

PSMA PET/CT significantly influences surgical decision-making, particularly for nerve-sparing radical prostatectomy. In a study by Bahler et al. authors aim was to determine if PSMA PET/CT in addition to mpMRI improved surgical decision making for non-nerve sparing or nerve sparing approach. Compared to mpMRI, surgeons were more likely to follow radiologists’ recommendations regarding organ confied disease when using PSMA PET/CT (adherence rate: 0.89 vs. 0.78, *p* < 0.1). In cases where findings differed regarding ECE, reliance on PSMA PET/CT led to correct surgical decisions, defined as selection of the nerve-sparing approach versus non-nerve sparing approach based on ECE, in 20 out of 27 cases, compared to only 7 with mpMRI alone (*p* = 0.01). In this study, 22 cases were altered from non-nerve-sparing to nerve-sparing prostatectomy without a significant increase in positive margin rates (25% vs. 24%, *p* = 0.89). This suggests that PSMA PET/CT can help preserve functional outcomes without compromising oncologic control [21].

### Lymph Node Assessment

A meta-analysis by Stabile et al. provided comprehensive data on PSMA PET/CT’s accuracy in detecting lymph node invasion (LNI) compared to pathology following extended pelvic lymph node dissection (ePLND) [22]. The pooled diagnostic accuracy showed an area under the curve (AUC) of 84%, with high specificity (93%) but moderate sensitivity (54%). Findings remained consistent in a secondary analysis including only studies with > 50 patients.

When stratified by risk group, sensitivity varied significantly (*p* = 0.008): 54% in high-risk, 93% in intermediate-risk, 58% in mixed-risk, and 56% in unspecified-risk cohorts. Specificity remained consistently high across all groups. The median prevalence of LNI was 26%, with PPV increasing from 59 to 91% as LNI prevalence rose from 5 to 40% (*r* = 0.69, *p* < 0.01), while NPV declined from 99 to 84% over the same range (*r* = 0.26, *p* = 0.037) [22].

Huebner et al. demonstrated that majority of patients with negative PSMA PET findings could safely avoid LND, reducing surgical morbidity without compromising oncologic outcomes [23, 24]. This is particularly relevant for intermediate-risk patients, where the high NPV of PSMA PET could help avoid unnecessary dissections.

## Emerging Applications

### Active Surveillance

The integration of PSMA PET/CT into active surveillance protocols represents a significant advancement in prostate cancer management, offering more precise risk stratification and monitoring capabilities. Its ability to detect clinically significant disease that may be missed by conventional imaging enhances patient safety and therapeutic decision-making, and potentially limits the number of surveillance biopies [25].

Beyond improving detection, PSMA PET/CT strengthens patient counseling and shared decision-making. Studies have demonstrated that patients who understand their disease status and imaging results are more likely to engage in their treatment plans [26]. This is particularly relevant in prostate cancer, where management options vary based on disease aggressiveness. By offering a clearer picture of tumor burden and distribution, PSMA PET/CT enables nuanced discussions about the risks and benefits of different strategies.

The clinical utility of PSMA PET/CT is reinforced by its strong correlation with histopathological outcomes. Studies have shown that it accurately predicts intraprostatic tumor foci, aiding in disease assessment and treatment planning [27]. Its high sensitivity and specificity in detecting prostate cancer have been well-documented, supporting its role in ongoing patient evaluation under active surveillance [28].

The incorporation of PSMA PET/CT into surveillance protocols also raises important considerations regarding cost-effectiveness and resource allocation. While the initial costs of advanced imaging are higher than traditional modalities, improved patient outcomes and reduced overtreatment could offset long-term healthcare expenditures. Economic evaluations have begun to assess the overall impact of PSMA PET/CT on healthcare utilization and patient quality of life [29].

### Treatment Response Monitoring

In the post-treatment setting, particularly for patients with BCR, PSMA PET/CT has become an invaluable tool for monitoring treatment response. Traditionally, clinicians relied on PSA kinetics to infer the nature of recurrence—whether local or systemic. However, PSMA imaging provides a more definitive assessment, enabling targeted interventions based on precise disease localization [30].

When PSMA PET/CT identifies a solitary bone lesion or lymph node involvement, treatment strategies such as stereotactic body radiotherapy (SBRT) can be considered, potentially delaying the need for systemic therapy [31]. Additionally, PSMA PET/CT facilitates surveillance in patients with advanced disease by detecting early progression, allowing for timely adjustments to treatment regimens.

The emergence of PSMA-targeted therapies, such as ¹⁷⁷Lu-PSMA radioligand therapy, represents a major advancement in the management of advanced prostate cancer. These therapies exploit the high expression of PSMA on tumor cells, delivering targeted radiation while sparing healthy tissue, thereby improving efficacy and reducing toxicity [32].

### Quantitative PSMA Assessment and Prognostication

PSMA imaging transcends traditional binary assessment, offering quantitative insights through standardized uptake values (SUV) that can guide clinical decision-making and patient counseling. Higher SUV values have been shown to correlate with more aggressive disease and increased likelihood of BCR [33, 34].

The quantification of PSMA uptake, often referred to as “SUVmax,” offers crucial insights into disease aggressiveness [35]. For example, patients with high SUVmax values may warrant more aggressive or multimodal treatment approaches, while those with lower values might be candidates for active surveillance or less morbid therapies [36]. This nuanced understanding enhances patient counseling and treatment planning across various risk categories [37].

The implications of PSMA imaging quantification extend to decisions regarding focal versus whole-gland therapy. Lesions exhibiting very high SUV values may indicate more aggressive disease biology, suggesting that these patients may be better suited for whole-gland or definitive therapy rather than focal treatment [38]. This is particularly relevant in localized prostate cancer, where treatment options can vary significantly based on biological characteristics.

The integration of quantitative PSMA imaging assessment necessitates a multidisciplinary approach, involving urologists, medical oncologists, and radiation oncologists, to ensure that treatment decisions are made collaboratively [39]. This collaborative framework is essential for optimizing patient outcomes and ensuring that treatment strategies align with the biological characteristics of the disease.

Furthermore, the dynamic assessment of SUV values over time can potentially provide valuable insights into treatment efficacy and disease status [40]. Patients undergoing PSMA-targeted therapies may experience changes in SUV values, which can guide decisions regarding the continuation or modification of treatment strategies [41].

## Limitations and Future Directions

Despite its revolutionary impact on prostate cancer management, PSMA imaging faces several important limitations that warrant careful consideration. First, the variability in PSMA expression among different prostate cancer subtypes can lead to false negatives, particularly in certain aggressive forms that may not exhibit significant PSMA expression [42, 43]. This biological heterogeneity necessitates careful patient selection for PSMA-based imaging and therapies.

Treatment-induced changes in PSMA expression present another challenge. Androgen deprivation therapy (ADT) and chemotherapy can modulate PSMA levels, potentially complicating the interpretation of imaging results and treatment response assessment [44]. This dynamic expression pattern raises important questions about optimal timing of imaging relative to treatment cycles.

False positives represent a third significant limitation. Benign conditions, particularly prostatic hyperplasia (BPH), hemangiomas, or fibrous dysplasia can exhibit elevated PSMA expression, potentially leading to unnecessary interventions or patient anxiety [42, 43]. Clinicians must develop expertise in distinguishing between malignant and benign causes of increased PSMA uptake.

The phenomenon of stage migration also merits careful consideration. PSMA PET/CT’s superior sensitivity in detecting small lesions has led to an apparent increase in early-stage diagnoses and previously unidentified metastases [45, 46]. This improved detection capability, while valuable, can complicate clinical decision-making and comparison with historical outcomes.

## Conclusion

The integration of PSMA imaging into prostate cancer management represents a significant paradigm shift in clinical practice. From initial staging to BCR and advanced disease, PSMA PET/CT has demonstrated superior performance compared to conventional imaging modalities. Its impact extends beyond simple disease detection, providing quantitative insights that inform treatment decisions and improve patient outcomes across the disease spectrum.

The role of PSMA imaging in local disease assessment, particularly in conjunction with mpMRI, has enhanced our ability to detect extracapsular extension and guide surgical planning. In the emerging applications of active surveillance and treatment response monitoring, PSMA imaging provides crucial information that enables more personalized approaches to patient care.

Despite its limitations, including variable expression patterns and the challenges of stage migration, PSMA imaging has fundamentally altered the landscape of prostate cancer management. As research continues to elucidate its full potential, particularly in quantitative assessment and novel therapeutic applications, PSMA imaging will likely play an increasingly central role in personalizing prostate cancer care.

Looking ahead, the continued refinement of PSMA-based techniques, combined with improved understanding of their optimal clinical implementation, promises to further enhance our ability to provide precise, personalized care for patients with prostate cancer. This evolution in imaging technology, coupled with expanding therapeutic applications, positions PSMA as a cornerstone in the future of prostate cancer management.

## Key References

- 1. Bahler CD, Tachibana I, Tann M, Collins K, Swensson JK, Green MA, Mathias CJ, Tong Y, Yong C, Boris RS, Brocken E, Hutchins GD, Sims JB, Hill DV, Smith N, Ferari C, Love H, Koch MO. Comparing Magnetic Resonance Imaging and Prostate-Specific Membrane Antigen-Positron Emission Tomography for Prediction of Extraprostatic Extension of Prostate Cancer and Surgical Guidance: A Prospective Nonrandomized Clinical Trial. J Urol. 2024 Aug;212(2):290–298. Epub 2024 May 24.
-
  - PSMA PET/CT may outperform MRI in predicting extraprostatic extension of prostate cancer, improving surgical planning by enhancing preoperative assessment and guiding nerve-sparing decisions.
- 2. Stabile A, Pellegrino A, Mazzone E, Cannoletta D, de Angelis M, Barletta F, Scuderi S, Cucchiara V, Gandaglia G, Raggi D, Necchi A, Karakiewicz P, Montorsi F, Briganti (A) Can Negative Prostate-specific Membrane Antigen Positron Emission Tomography/Computed Tomography Avoid the Need for Pelvic Lymph Node Dissection in Newly Diagnosed Prostate Cancer Patients? A Systematic Review and Meta-analysis with Backup Histology as Reference Standard. Eur Urol Oncol. 2022 Feb;5(1):1–17. doi: 10.1016/j.euo.2021.08.001. Epub 2021 Sep 17. PMID: 34,538,770.
-
  - A negative PSMA PET/CT may help avoid unnecessary pelvic lymph node dissection in newly diagnosed prostate cancer patients by reliably ruling out metastatic lymph node involvement.
- 3. J Huebner NA, Wasinger G, Rajwa P, Resch I, Korn S, Rasul S, Baltzer P, Prüger L, Rauschmeier A, Seitz C, Comperat E, Shariat SF, Grubmüller (B) Clinical parameters for the prediction of occult lymph node metastasis in patients with negative PSMA-PET. Urol Oncol. 2024 Apr;42(4):115.e9-115.e16. doi: 10.1016/j.urolonc.2023.12.016. Epub 2024 Jan 20. PMID: 38,246,806.
-
  - This study identified clinical predictors of occult lymph node metastasis in patients with negative PSMA-PET, aiding in risk stratification and treatment decisions in urologic oncology.

## Data Availability

No datasets were generated or analysed during the current study.
